# Biomass Accumulation and Carbon Sequestration in Four Different Aged *Casuarina equisetifolia* Coastal Shelterbelt Plantations in South China

**DOI:** 10.1371/journal.pone.0077449

**Published:** 2013-10-15

**Authors:** Faming Wang, Xin Xu, Bi Zou, Zhihua Guo, Zhian Li, Weixing Zhu

**Affiliations:** 1 Key Laboratory of Vegetation Restoration and Management of Degraded Ecosystems, South China Botanical Garden, Chinese Academy of Sciences, Guangzhou, PR China; 2 Institute of Wetland Research, Chinese Academy of Forestry, Beijing, PR China; 3 Department of Biological Sciences, State University of New York-Binghamton, Binghamton, New York, United States of America; Tennessee State University, United States of America

## Abstract

Thousands of kilometers of shelterbelt plantations of *Casuarina equisetifolia* have been planted to protect the southeast coastline of China. These plantations also play an important role in the regional carbon (C) cycling. In this study, we examined plant biomass increment and C accumulation in four different aged *C. equisetifolia* plantations in sandy beaches in South China. The C accumulated in the *C. equisetifolia* plant biomass increased markedly with stand age. The annual rate of C accumulation in the *C. equisetifolia* plant biomass during 0–3, 3–6, 6–13 and 13–18 years stage was 2.9, 8.2, 4.2 and 1.0 Mg C ha^−1^ yr^−1^, respectively. Soil organic C (SOC) at the top 1 m soil layer in these plantations was 17.74, 5.14, 6.93, and 11.87 Mg C ha^−1^, respectively, with SOC density decreasing with increasing soil depth. Total C storage in the plantation ecosystem averaged 26.57, 38.50, 69.78, and 79.79 Mg C ha^−1^ in the 3, 6, 13 and 18- yrs plantation, with most of the C accumulated in the aboveground biomass rather than in the belowground root biomass and soil organic C. Though our results suggest that *C. equisetifolia* plantations have the characteristics of fast growth, high biomass accumulation, and the potential of high C sequestration despite planting in poor soil conditions, the interactive effects of soil condition, natural disturbance, and human policies on the ecosystem health of the plantation need to be further studied to fully realize the ecological and social benefits of the *C equisetifolia* shelterbelt forests in South China.

## Introduction

Forest restorations, including tree plantations, are often proposed as a remedy to combat global climate change. Globally, plantations are being established at an increasing rate, and now accounting for 5% of the global forest cover [1]. Trees can capture atmospheric CO_2_ through photosynthesis and store it in biomass with a turnover time of several decades. Thus, tree plantations play important roles in global C cycling and the uses of tree products can mediate various anthropogenic C releases [2], [3]. China maintained the largest plantation area in the world, reaching 62 million ha in 2008, and accounting for 35.5% of the total forest area in China [4]. Shelterbelt plantation, established along coastlines, is a special type of well-protected plantation in China due to socio-economic considerations. There is a “zero tolerance” policy on illegal act of cutting and tree harvesting, although local collecting of litter and fallen dead trees are often allowed. China has 18,000 kilometers mainland coastline, corresponding to 11.3 million hectares of coastal land; this provides a wide range of land resource for coastal shelterbelt plantation.

*Casuarina equisetifolia* is an N-fixing species that has been used extensively for windbreak and coastal stabilization in tropical and sub-tropical areas of the world [5], [6]. The critical ecosystem services of coastal forests, including *Casuarina* plantations, have gained great recognition recently, particularly after the devastating 2004 Southeast Asian tsunami [6], [7]. *Casuarina equisetifolia* is naturally distributed in the Oceania, Pacific islands and Southeast Asia [8], [9]. It requires limited growth conditions, likely because of its actinorhizal and mycorrhizal symbioses that fix nitrogen (N) and benefit phosphorus (P) acquisition [9], [10]. Since 1950 s, *C. equisetifolia* has been planted in the southeast coast of China [11]. The area of the *C. equisetifolia* plantation in China has currently reached 300,000 ha, with various field and nursery experimental trials been conducted [10], [12].

*Casuarina equisetifolia* plantation has the potential to sequestrate atmospheric CO_2_ and contributes to the regional C cycling. Most studies have focused on the ecological functions of *C. equisetifolia*, such as land reclamation, windbreaks, erosion control, and wood and fuel production [5], [6]. Many N-fixation studies have been done on this plant [13], [14]. In addition, its arbuscular mycorrhizal (AM) and ectomycorrhizal (ECM) symbioses have also been examined [10], [15]. However, the aboveground and belowground biomass increment and C sequestration in *C. equisetifolia* plantations in South China are largely unknown. Due to its large actual and potential planting areas in China and other parts of the world, the C sequestration potential in *C. equisetifolia* may greatly account for the regional and global C budget.

The patterns of C stock during forest development have gained great attention since one century ago [16]. Many studies have reported changes in biomass and production with stand age using chronosequence method [17]. Generally, plant biomass would increase gradually with stand age. However, soil C might perform differently. The decomposition of soil C might proceed more rapidly than the plant C input in the initial stage, resulting in a net loss of soil C at early stage of stand development. In this paper, we investigated the plant biomass increment and soil C change in four different aged *C. equisetifolia* plantations growing at a sandy beach site in South China. The objectives of this study were to: 1) quantify aboveground and belowground biomass of *C. equisetifolia* at different aged plantations; 2) estimate C sequestration in plant biomass and soil. Our hypotheses were that (1) ecosystem C storage (including biomass C and soil C) would increase with the stands ages; and (2) soil might contribute to carbon storage less than plant biomass as a result of unfavorable texture of sandy soil.

## Materials and Methods

### Ethics Statement

This research was conducted in South China Botanical Garden, Chinese Academy of Sciences. This study was also supported by this institute. We confirmed that the location is not privately-owned and the sampling of soils and plants was approved by Forestry Agency of Maogang District, the local administrator of coastal shelterbelt plantations. We also confirmed that the field studies did not involve endangered or protected species.

### Site Description

The study area is located in the coastal Maogang District, southeast of Maoming City (110°54′E, 21°27′N), Guangdong Province, South China. The region has a tropical monsoon climate, including a rainy and warm season (April to October: precipitation 1400 mm) and a dry and cool season (November to March: precipitation 160 mm). Typhoons occur in this area from June to late October. The annual mean temperature is 23°C, with an average July high of 30°C and January low of 18°C.

*Casuarina equisetifolia* has been planted continuously in this area since 1960 s. Due to the disturbance of typhoon, the plantations were frequently destroyed and replanted again. However, as a substitute for fuel material, the litterfalls of *C. equisetifolia* were collected regularly by the local residents, so were the fallen trees after the typhoon disturbance.

### Experiment Design

We applied a chronosequence approach. Four different age classes of *C. equisetifolia* plantations were selected, which were established in 1994, 1999, 2006 and 2009, respectively. Soil texture was very homogenous in these plantations, with sand accounting for 92–93% and clay for 4–5% of the soil mass (Table S1). The original number of trees planted at all sites is 2500 plant ha^−1^. Four 10 m×10 m plots were established in each of the four plantations in March 2012. Species density, tree height (H) and tree diameter at breast height (DBH) were recorded at each plot (Table 1). We used a *C. equisetifolia* growth model developed specifically for the coastal South China region to estimate the plant biomass [18]. Plant biomass included root, stem, branch, and branchlet was estimated in each plot (Table S2).

**Table 1:** The general status of four age classes of *C. equisetifolia* plantations in 2011 (mean±S.E.).

Stand age(yrs)	Density(plants ha^−1^)	DBH(cm)	Height(m)
3	2350^a^±87	4.49^d^±0.28	4.92^c^±0.63
6	2200^a^±82	9.46^c^±0.14	8.16^b^±0.40
13	1250^b^±144	15.88^b^±0.40	12.67^a^±0.16
18	975^b^±63	19.35^a^±0.90	10.63^b^±0.57

Note: DBH: Diameter at breast height. Means in a column followed by different lower-case letters are significantly different at *P*<0.05 (one-way ANOVA and LSD test).

### Field Sampling and Measurements

We collected samples of *C. equisetifolia* branchlets, branches, stems and fine roots at each individual plot, and analyzed each component separately for their relative concentration of C. Samples were dried to a constant weight at 65°C, ground and passed through a fine screen (0.5 mm). The C concentration was determined using the potassium dichromate oxidation method [19], and then applied to the growth model to estimate biomass C accural.

Roots were also sampled directly using a 25 cm diameter PVC pipe. Root cores were taken at 3 random places in each plot at the 0–10, 10–20, 20–40, 40–60, 60–80 and 80–100 cm soil depths. Roots were washed free of soil with a spray of water. Fine root (<2 mm) was separated from coarse root (>2 mm) and dried at 65°C to a constant weight.

Soil samples were collected from 3 positions in each plot by using a 5 cm diameter PVC tube at the 0–10, 10–20, 20–40, 40–60 and 60–100 cm soil depths. Bulk density was determined by taking an adjacent core. All of soil samples within the same soil depth in each plot were pooled together and air-dried. Before analyzing total N (TN) and organic matter content, soils were ground to pass through a 0.25-mm sieve. Total N concentration was determined by the micro-Kjeldahl digestion followed by the colorimetric determination [19] on a flow injection autoanalyzer (FIA) (Lachat Instruments, USA). Soil organic carbon (SOC) was determined by the potassium dichromate oxidation method with SOM calculated as SOM = 1.724×SOC [19].

### Statistical Analysis

Statistical analyses were performed using SAS 8.1 for Windows (SAS Institute, Cary, NC, USA.). Biomass accumulation, carbon concentrations and C storage of plant materials and soils, as well as fine root distribution among 4 age classes of plantations were compared by one-way ANOVA, followed by the LSD method to test among different age groups.

## Results

### Plant Biomass and C Concentration

Aboveground biomass (AGB), belowground biomass (BGB), and total plant biomass (PB) of the *C. equisetifolia* plantations increased markedly with stand age. The plantation followed a classic self-thinning growth with the steady increases of DBH and height along with the decrease of tree density (Table 1). The AGB and BGB of *C. equisetifolia* plantation increased from 14.22 and 7.77 Mg ha^−1^ at the 3 yrs-old plots to 130.34 and 21.13 Mg ha^−1^ at the 18 yrs-old plots, respectively (Table 2). The PB of *C. equisetifolia* plantation in our study site increased from 21.99 Mg ha^−1^ at the 3 yrs-old plots to 151.46 Mg ha^−1^ at the 18 yrs-old plots, with the most rapid increase occurred in the 3–6 years stage (Table 2). The biomass of stem and root accounted for more than 70% of the total plant biomass. In the four age classes plantations, we found that the proportion of stem biomass was the largest and the branchlet biomass was the smallest. The BGB/AGB ratio was 0.61, 0.21, 0.16, and 0.16 at the 3, 6, 13, and 18 yrs old plantation, respectively.

**Table 2:** Biomass of four different age classes of *C. equisetifolia* plantations (Mg ha^−1^, mean ± S.E.).

Stand age(yrs)	Stem	Branch	Branchlet	AGB	Root (BGB)	PB
3	8.80^c^±2.06	2.59^c^±0.52	2.83^b^±0.37	14.22^c^±2.96	7.77^c^±0.43	21.99^c^±3.33
6	46.05^b^±2.79	10.65^b^±0.61	7.01^a^±0.35	63.71^b^±3.74	13.1^b^±0.60	76.9^b^±4.32
13	96.0^a^±6.70	18.3^a^±1.35	8.12^a^±0.69	122.5^a^±8.69	19.6^a^±1.44	142.1^a^±10.1
18	104.6^a^±21.9	18.5^a^±3.31	7.20^a^±0.91	130.3^a^±26.1	21.1^a^±4.32	151.5^a^±30.4

Note: AGB refers to aboveground biomass, PB refers to total plantation biomass. Means in a column followed by different lower-case letters are significantly different at *P*<0.05 (one-way ANOVA and LSD test).

There was significant difference in the C concentrations among different parts of *C. equisetifolia* tree (*P*<0.05, data not shown). Across all age groups, stems had the highest C concentration with mean value of 45.69%. In contrast, fine roots had the lowest C concentration with a mean of 35.32%. The mean C concentration of branches and branchlets was 45.28% and 42.08%, respectively.

### Soil C, N Concentration and Fine Root Distribution

**Figure 1:**
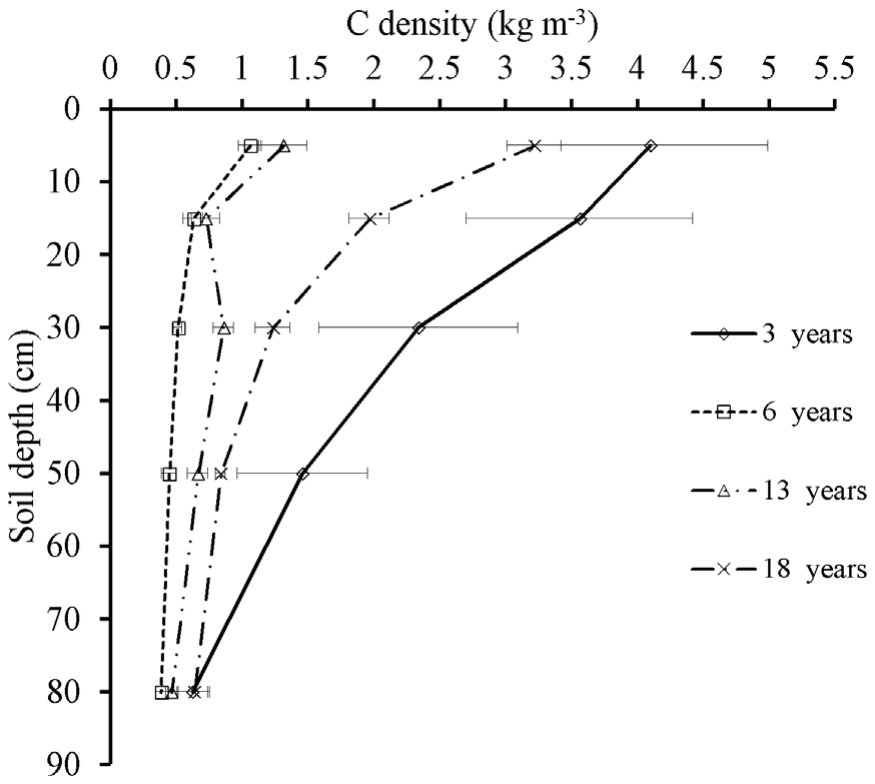
The soil carbon density of four different age classes of *C. equisetifolia* plantations. Note: Error bar indicating SE.

In the 0–10 cm soil, mean SOC concentrations ranged from 0.71 g kg^−1^ in the 6-yrs plots to 2.67 g kg^−1^ in the 3-yrs plots (Table 3). In all age plantations, SOC decreased with increasing soil depth (Fig. 1). There was a significant difference in the SOC density among the four age classes *C. equisetifolia* plantations respectively (*P*<0.05, Fig. 1), with the highest values in 3-yrs plots and lowest ones in 6-yrs plots in all soil layers. Soil total N (TN) was also low in these plantations. In the 0–10 cm soil, TN ranged from just 0.09 g kg^−1^ in the 6-yrs plots to 0.39 g kg^−1^ in the 3-yrs plots. Soil TN followed a similar decline with increasing soil depth like the SOC (Table 3). Like SOC, TN was highest in the 3-yrs plots, declined to the lowest concentration in the 6-yrs plots, and then gradually recovered in the 18-yrs plots. C/N ratios of the 0–10 cm soils ranged from 6.94 to 11.33, and the ratios typically decreased with increasing soil depth (Table 3).

**Table 3:** Properties of the soil in four different age classes of *C. equisetifolia* plantations (only 0–10 and 10–20 cm soil data were presented here, mean ± S.E.).

Variables			Stand age		
	Soildepth	3	6	13	18
BulkDensity(g cm^−3^)	0–10 cm	1.54±0.02	1.50±0.01	1.49±0.03	1.53±0.02
	10–20 cm	1.50±0.02	1.50±0.01	1.47±0.01	1.48±0.02
SOC(g kg^−1^)	0–10 cm	2.67^a^±0.59	0.71^b^±0.06	0.87^b^±0.11	2.10^a^±0.14
	10–20 cm	2.35^a^±0.55	0.43^c^±0.05	0.49^c^±0.07	1.31^b^±0.11
TN (g kg^−1^)	0–10 cm	0.39^a^±0.02	0.09^c^±0.01	0.13^c^±0.02	0.19^b^±0.02
	10–20 cm	0.32^a^±0.04	0.09^c^±0.01	0.09^b^±0.02	0.15^b^±0.02
C/N	0–10 cm	6.94^b^±1.66	7.62^b^±0.68	6.68^b^±0.27	11.33^a^±1.38
	10–20 cm	7.12^ab^±1.39	4.84^c^±0.59	5.85^bc^±0.62	9.44^a^±1.37

Note: Means in a row followed by different lower-case letters are significantly different at P<0.05 (one-way ANOVA and LSD test).

Mean fine root biomass at 1 m soil depth averaged 2.62, 4.48, 2.69 and 2.82 Mg ha^−1^ in the 3, 6, 13 and 18 yrs old *C. equisetifolia* plantations, respectively (Table 4). Six yrs plantation had the highest fine root biomass among the four age classes plantations (*P*<0.05). The fine root biomass decreased with an increase in soil depth, with a large proportion located in the top 40 cm soil (70.1%, 46.7%, 62.8%, and 78.9% of the total in the 3-yrs, 6-yrs, 13-yrs, and 18-yrs old plantations, respectively). Fine roots were sparse in the 3-yrs old *C. equisetifolia* plantation, especially in the deep soil; then it increased in the 6-yrs old plantation and declined again in the 13- and 18-yrs plantation (Table 4), despite the increase of total plant biomass over time.

**Table 4:** The depth distribution of fine root biomass at four age classes of *C. equisetifolia* plantations (Mg ha^−1^, mean ± S.E.).

Stand age (yrs)	0–10 cm	10–20 cm	20–40 cm	40–60 cm	60–80 cm	80–100 cm	Total
3	0.68±0.09	0.54±0.06	0.62±0.09	0.34±0.12	0.29^b^±0.13	0.16^b^±0.07	2.62^b^±0.37
6	0.59±0.04	0.64±0.11	0.86±0.27	0.80±0.09	0.65^a^±0.13	0.94^a^±0.22	4.48^a^±0.40
13	0.59±0.20	0.40±0.10	0.71±0.16	0.41±0.06	0.23^b^±0.08	0.36^b^±0.12	2.69^b^±0.55
18	1.03±0.25	0.55±0.06	0.64±0.23	0.38±0.19	0.09^b^±0.04	0.13^b^±0.06	2.82^b^±0.67

Note: Means in a column followed by different lower-case letters are significantly different at *P*<0.05(LSD test).

### Ecosystem C Sequestration

Most plant C was accumulated in the aboveground biomass, especially in stems. Carbon storage in the aboveground plant biomass averaged 6.3, 28.8, 55.9, and 59.7 Mg C ha^−1^ in the 3, 6, 13, and 18 yrs old plantations respectively (Table 5). Root biomass C increased from 2.5 Mg C ha^−1^ in the 3-yrs plots to 8.3 Mg C ha^−1^ in the 18-yrs plots, representing 28.2%, 13.7%, 11.0%, and 12.2% of the total plant biomass C in the four age classes of plantations. The C accumulated in the *C. equisetifolia* plant biomass increased markedly with stand age. The annual rate of C accumulation in the *C. equisetifolia* plant biomass during 0–3, 3–6, 6–13 and 13–18 yrs stage was 2.9, 8.2, 4.2 and 1.0 Mg C ha^−1^ yr^−1^, respectively (Table 6).

**Table 5:** Carbon storage in biomass and soils in four *C. equisetifolia* plantations (Mg ha^−1^, mean ± S.E.).

Stand age(yrs)	Stem	Branch	Leaf	AGCS	Root	TBCS	SOC	TCS
3	3.94^c^±0.92	1.18^c^±0.24	1.22^b^±0.16	6.34^c^±1.32	2.49^c^±0.14	8.83^c^±1.44	17.74^a^±3.21	26.57^c^±2.80
6	21.04^b^±0.00	4.82^b^±0.27	2.93^a^±0.14	28.78^b^±0.42	4.58^bc^±0.21	33.37^b^±0.63	5.14^c^±0.24	38.51^b^±2.11
13	44.19^a^±3.09	8.34^a^±0.61	3.41^a^±0.29	55.94^a^±3.97	6.91^ab^±0.51	62.85^a^±4.48	6.93^c^±0.24	69.78^a^±4.68
18	48.35^a^±10.1	8.31^a^±1.49	2.99^a^±0.38	59.65^a^±12.0	8.26^a^±1.69	67.91^a^±13.6	11.87^b^±0.76	79.78^a^±13.91

Note: SOC, TBCS, AGCS and TCS refer to soil organic carbon (0–100 cm), total biomass C storage, aboveground C storage and total C storage, respectively. Means in a column followed by different lower-case letters are significantly different at *P*<0.05(one-way ANOVA and LSD test).

**Table 6:** Average annual rate of biomass carbon accumulation at four age classes of *C. equisetifolia* plantations (Mg C ha^−1 ^yr^−1^).

Stand stage	AGC	BGC	TBC
0–3 yrs	2.1	0.8	2.9
3–6 yrs	7.5	0.7	8.2
6–13 yrs	3.9	0.3	4.2
13–18 yrs	0.7	0.3	1.0

Note: AGB refers to aboveground biomass C; BGC refers to belowground biomass C; TBC refers to total biomass C.

Soil organic C storage in the top 100 cm soil was 17.74, 5.14, 6.93, 11.87 Mg ha^−1^ at the 3, 6, 13 and 18 yrs plantation, respectively (Table 5). The highest soil carbon storage existed at the 3 yrs plantation and the lowest at the 6 yrs plantation, with SOC storage increased markedly from 6-yrs plots to 13-yrs and 18-yrs plots (Table 5).

Total C storage in the plantation ecosystem averaged 26.57, 38.50, 69.78, and 79.79 Mg C ha^−1^ in the 3, 6, 13 and 18- yrs plantation respectively, with most of the C accumulated during the 18 yr growth stored in the aboveground biomass rather than in the belowground root biomass and soil organic C (Table 5).

## Discussion

### Biomass C Accumulation in *Casuarina Equisetifolia* Plantations

This study supports our hypothesis that *C. equisetifolia* biomass and C storage increased quickly with plantation age in both aboveground and belowground parts. A number of studies have reported similar trends of forest growth [20], [21]. Moreover, the accumulation rate was fast in the early stages of plantation and then slowed down as the plantation aged. The highest accumulation rate was observed in the 3–6 yrs old stage (Table 6). The biomass of the fine root also reached maximum values in this stage (Table 4). These results were consistent with other studies, which found the largest fine root biomass in the fast-growth stage stand [22], [23].

The *C. equisetifolia* plantation also accumulated more biomass than many other secondary tropical forests. Harmand et al. [24] reported that total biomass in the 7 yrs old *Eucalyptus camaldulensis* and 6 yrs old *Senna siamea* was 62.35 and 45.39 Mg ha^−1^, respectively. Similarly, aboveground biomass in the 7 yrs old secondary forests in the Uxpanapa Region of Veracruz, Mexico was 52.7 Mg ha^−1^ [25]. Miao et al. [26] examined biomass of different mangrove forests in South China and found that total forest biomass in the 5 yrs old *Aegiceras corniculatum*, *Avicennia marina*, and *Kandelia candel* forests was 5.5, 16.4, and 62.6 Mg ha^−1^, respectively. These values were smaller than the 63.71 Mg ha^−1^ (6 yrs old plantation) found in our study. Our data thus suggest that the *C. equisetifolia* plantations can accumulate large amount of biomass in both of aboveground and belowground parts, despite growing in the sandy coastal soils with low nutrient and low organic matter.

Most tropical forests accumulate large amounts of biomass in their roots [24], [26]. In this study, however, we found average BGB (Belowground biomass)/AGB (Aboveground biomass) ratio was just 0.27 in the four *C. equisetifolia* plantations. The ratios reported here was much lower than those reported in other tropical forests. Harmand et al. [24] reported that the BGB/AGB ratio was 0.76, 0.90, and 0.44 in the 5 yrs old *Acacia polyacantha, S. siamea* and *E. camaldulensis* forests, respectively. However, the average BGB/AGB ratio was 0.29 in the humid tropical forests in Costa Rica [27], a value much closer to ours. In the mangrove forests, Miao et al. [26] reported that BGB/AGB ratio for the three 5 yrs old mangrove forests was 0.6, 0.7, and 0.8, respectively.

### Soil Organic C and TN in *Casuarina Equisetifolia* Plantations

Our study did not support the hypothesis that C storage in the soil increased with plantation age. Davis et al. [28] reported an increase of SOC from 29.8 Mg ha^−1^ to 42.0 Mg ha^−1^ in the mineral soil (0–10 cm) along a stand development sequence in a New Zealand *Nothofagus* forest. Sartori et al. [29] conducted a chronosequence study of poplar plantations in USA. They found that C concentration in the upper 5 cm of the mineral soil increased with plantation age but decreased with age at 5–15 cm and 15–25 cm depths. Similar results had been reported in other studies [30], [31]. In our study, SOC decreased in the early stage after the reforestation and then gradually increased with the stand age. Paul et al. [32] showed the similar result in an afforestation site in Australia. They found that surface soil (<10 or <30 cm depth) C generally decreased during the first 5 years but then increased, and recovered after about 30 yrs afforestation. It is well known in ecological literature that during early stages of forest development, fast litter decomposition and SOC mineralization could lead to SOC decrease (before its eventual increase), a phenomena termed “Covington curve” [33], [34]. Such initial decrease of SOC followed by SOC increase have been shown in many other studies [35]–[38].

Land use history had a significant effect on changes in soil C, so had the soil disturbance during the site preparation for forest growth [20], [39], [40]. In the first several years of reforestation, there is relatively little input of C from aboveground [41]. However, C from residues of the preceding plantation continues to decompose during this time. In addition to the land use history, the methods of cultivation and management also have significant effects on the C content [32]. During many plantation site preparations in China, soils were often heavy disturbed, sometimes including root excavation, which can greatly accelerate SOC loss in the early years of reforestation.

The litterfalls of *C. equisetifolia* were collected regularly by the local residents as biofuel materials. As an important aboveground C input into soils, the harvesting of litter usually results in additional losses of C from the soil. Many studies have suggested that retaining residues on site could reduce soil C loss [42], and the removing or burning of litters and residues always lead to a large loss of C. Thus, our study suggests that given appropriate management practices (i.e., retaining litterfall), the *C. equisetifolia* plantations would have a higher potential to sequester more C.

Nitrogen is one of the most common limiting elements in terrestrial ecosystems that limit the primary production and other ecological processes [43], [44]. Soil N would vary considerably during soil development as N accumulates through N-fixation and deposition [45]. In our study, the concentration of soil TN ranged from 0.09 to 0.39 g kg^−1^ in the 0–10 soil layer, which was much lower than the data reported in nearby soils. For example, Mo et al. [46] reported that total N of top soil (0–20 cm) in the disturbed, rehabilitated and mature forests in tropical China was 0.9, 1.0, and 1.9 g kg^−1^, respectively. The specific soil type may partially explain the low N status in this study. More than 92% of sand fraction of the soil might enhance the soil N loss through leaching during N mineralization processes. In addition, the collection of litterfall by the local residents might be another reason for the low concentration in soil N, because litterfall is a major pathway of nutrient return to the soil [47].

### C Accrual in *Casuarina Equisetifolia* Plantations

Our results suggested that organic C can accumulate rapidly in the *C. equisetifolia* plantations in South China. Furthermore, most C accrual was due to high biomass accumulation. The estimation of annual biomass C accumulation rate of *C. equisetifolia* plantation reached 8.2 Mg/ha in 3–6 yrs stage (Table 6). This value was higher than the data in 3 yrs old *A. crassicarpa* plantation (6.5 Mg/ha) in tropical China [48]. Our estimation was also consistent with previous study [49]. Yang and Guan [49] have reported annual biomass C accumulation rate in various forests of Pearl River Delta, and found that *C. equisetifolia* plantation (7.25 Mg/ha) had the fastest rate among different forest types (ie: *Pinus elliottii* plantation: 4.8 Mg/ha, Acacia plantation: 4.7 Mg/ha, broadleaf forests: 6.5 Mg/ha).

In consistent with our hypotheses, the contribution of SOC to total ecosystem C storage decreases with the stand age. SOC was 17.74, 5.14, 6.93, and 11.87 Mg C ha^−1^ at the 3, 6, 13 and 18 yrs old plantation, respectively, representing 66.8%, 13.3%, 9.9% and 14.9% of the total ecosystem carbon. The percentage was smaller than many other studies. Fonseca et al. [27] reported that the amount of carbon stored in the soil represented 74.3% of the total carbon in forest, 51.5% higher than the biomass C in the humid tropics of Costa Rica. The rapid accrual of ecosystem C in the *C. equisetifolia* plantations has benefited from the coastal protection policy of “zero tolerance” on tree cutting and harvesting. Conversely, the low soil nutrient (TN) and SOC content in the coastal sandy soil may limit soil microbial mediated nutrient turnover, subsequently, may limit the C accumulation potential of this ecosystem. The C accumulation potential is further constrained by the routine litter removal by local residents. The litter removal not only reduced direct SOC flow belowground, but also diminished essential soil nutrients (N, P) for plant growth and further C accumulation in the system.

Since there is a large planting area reaching 300,000 ha for this species in the coastal area of China [12], *C. equisetifolia* plantations might have played great roel in sequestering C. To fully realize such potential, we need to understand the interactive effects of soil nutrient, natural disturbance, and human decisions on the ecosystem health of the *C. equisetifolia* plantations. The large-scale *Casuarina* plantations as shelterbelt forest along the coastline in South China has important roles beyond C sequestration, including restoring degraded coastal land and protecting coastal community, thus the social and ecological factors that affecting such critical ecosystem services need to be further investigated.

## Conclusions

*Casuarina equisetifolia* plantations (3–18 years old) in this study could rapidly accumulate large quantities of biomass. The total plant biomass of *C. equisetifolia* plantation at 3, 6, 13, and 18 yrs old plantations was 22.0, 76.8, 142.1, and 151.5 Mg ha^−1^, respectively, greater than many other tropical forests of similar ages. The SOC content was 17.74, 5.14, 6.93, and 11.87 Mg ha^−1^, representing 66.8%, 13.3%, 9.9% and 14.9% respectively of the total ecosystem carbon pool. Our study suggests that these plantations have a greater potential to sequestrate C despite poor soil conditions. The expansion of *C. equisetifolia* plantations in the South China can play important roles in the regional C budget and coastal protection. Long-term monitoring and research are needed to further explore the ecological and social-economic factors that affect the C sequestration and ecosystem health of these shelterbelt forests.

## Supporting Information

Table S1 Soil physical properties in the four age classes *C. equisetifolia* plantations.
(DOCX)

Table S2 The relative growth equations for *C. equisetifolia* plantations.
(DOCX)
