# Supplementary material for: Biomass Accumulation and Carbon Sequestration in Four Different Aged Casuarina equisetifolia Coastal Shelterbelt Plantations in South China
Source: PLoS One. 2013 Oct 15;8(10):e77449. doi: 10.1371/journal.pone.0077449 (PMC3797117; doi:10.1371/journal.pone.0077449)
Supplement: Table S1 — Soil physical properties in the four age classes C. equisetifolia plantations. (DOCX) [file pone.0077449.s001.docx]

Table S1 Soil physical properties in the four age classes *C. equisetifolia* plantations

| Stand age  （yrs） | Soil depth | BD | Soil texture（%） | | |
| --- | --- | --- | --- | --- | --- |
|  | (cm) | （g/cm^3^） | Clay | Silt | Sand |
| 3 | 0-10 | 1.54±0.02 | 3.7±1.18 | 3.97±1.52 | 92.33±0.62 |
|  | 10-20 | 1.50±0.02 | 4.5±1.23 | 1.43±0.84 | 94.07±0.80 |
|  | 20-40 | 1.50±0.04 | 2.78±0.88 | 4.15±0.92 | 93.07±0.66 |
|  | 40-60 | 1.51±0.02 | 3.79±0.51 | 2.04±0.33 | 94.17±0.22 |
|  | 60-100 | 1.51±0.02 | 3.87±0.78 | 2.65±0.32 | 93.48±0.93 |
|  | Mean | 1.51^a^±0.01 | 3.73±0.63 | 2.85±0.57 | 93.42±0.14 |
| 6 | 0-10 | 1.50±0.01 | 4.11±0.84 | 1.48±0.61 | 94.41±1.10 |
|  | 10-20 | 1.47±0.01 | 6.70±1.05 | 1.72±1.06 | 91.58±1.99 |
|  | 20-40 | 1.45±0.03 | 2.63±1.70 | 2.23±0.96 | 95.14±1.11 |
|  | 40-60 | 1.43±0.03 | 3.77±0.81 | 2.94±1.97 | 93.29±2.15 |
|  | 60-100 | 1.39±0.02 | 5.17±0.81 | 0.93±0.36 | 93.90±0.79 |
|  | Mean | 1.45^b^±0.02 | 4.48±0.74 | 1.86±0.47 | 93.66±1.02 |
| 13 | 0-10 | 1.49±0.03 | 4.29±1.14 | 2.07±0.92 | 93.64±0.74 |
|  | 10-20 | 1.48±0.02 | 4.44±1.49 | 2.67±1.34 | 92.89±0.31 |
|  | 20-40 | 1.46±0.01 | 5.37±1.32 | 1.92±0.75 | 92.71±0.69 |
|  | 40-60 | 1.44±0.02 | 4.85±1.80 | 2.37±1.16 | 92.78±0.74 |
|  | 60-100 | 1.46±0.02 | 5.72±1.51 | 2.57±0.97 | 91.71±0.93 |
|  | Mean | 1.47^b^±0.01 | 4.93±1.18 | 2.32±0.71 | 92.75±0.53 |
| 18 | 0-10 | 1.53±0.02 | 4.07±0.41 | 5.92±3.83 | 90.01±3.85 |
|  | 10-20 | 1.50±0.02 | 3.97±0.47 | 1.91±0.41 | 94.12±0.76 |
|  | 20-40 | 1.51±0.01 | 3.59±0.55 | 2.77±1.03 | 93.64±1.11 |
|  | 40-60 | 1.52±0.01 | 5.19±1.15 | 4.27±3.52 | 90.54±2.92 |
|  | 60-100 | 1.51±0.01 | 4.44±0.48 | 1.62±0.64 | 93.94±1.05 |
|  | Mean | 1.51^a^±0.01 | 4.25±0.47 | 3.30±1.07 | 92.45±1.27 |

Note: Mean is the average value of 0-100 cm soils. The labelled different uppercase letters on mean indicated significant difference（LSD: *P*<0.05）
