# Supplementary material for: Biomass Accumulation and Carbon Sequestration in Four Different Aged Casuarina equisetifolia Coastal Shelterbelt Plantations in South China
Source: PLoS One. 2013 Oct 15;8(10):e77449. doi: 10.1371/journal.pone.0077449 (PMC3797117; doi:10.1371/journal.pone.0077449)
Supplement: Table S2 — The relative growth equations for C. equisetifolia plantations. (DOCX) [file pone.0077449.s002.docx]

Table S2 The relative growth equations for *C. equisetifolia* plantations. (From Hong et al. 2010)

| Components | Equation | r^2^ | F | P-values |
| --- | --- | --- | --- | --- |
| Root | W=2.812+0.004(D^2^H) | 0.940 | 486.029 | <0.001 |
| Stem | W=0.045(D^2^H)^0.922^ | 0.944 | 525.915 | <0.001 |
| Branch | W=0.027(D^2^H)^0.781^ | 0.706 | 74.531 | <0.001 |
| Branchlet | W=0.122(D^2^H)^0.494^ | 0.602 | 46.806 | <0.001 |

Note: D: Diameter at breast height; H: Tree Height.
